# St. Bartholomew's Hospital

**Published:** 1833-02

**Authors:** 


### ST. BARTHOLOMEW'S HOSPITAL.

### *Ptyalism, with extensive Swelling of the Tongue, from the use of Mercury.*

Emma Lewis, æt. twenty-four, was admitted into Magdalene ward, under Mr. Lawrence, November 8th, with a discharge from the vagina, condylomatous swelling of the labia and thigh, attended with syphilitic ulceration of the tongue, and sore throat. She was ordered: ℞. Pil. Hydrarg. comp. nocte maneque s.; Extract. Sarsæ ℥ss. ter in dies adhibend. Lotio Zinci Sulphatis pro inject. in vaginam injiciend.

11. In consequence of pain in the head, twelve leeches were applied to the temples.—Sumat Pulv. Jalap. c. calomel.

12. Very little relief; mouth somewhat affected by the mercury; the right eye seems rather inflamed.—Rep. hirud. xij. ad tempora applicand.

13. The inflammation of the eye still remaining, with pain in the head; she was cupped on the right temple to sixteen ounces and ordered—Nitratis Argenti, gr. iv.; Aquæ distil. ℥j. M. ft. lotio; to be dropped into the eye. In this state she continued, the syphilitic disease of the labia and tongue having in a measure subsided, until the 12th, but in consequence of the great pain in the head, and inflamed appearance of the eye, she was ordered on

19. Emp. lyttæ inter scapulas.

20. C.c. ad ℥xx. nuchæ.

December 4. Hirud. xvj. around the eyebrow, in consequence of severe aching pain in that part, and on

10. Emplast. lyttæ ad nuch., and c.c. templo dextro. At this time there were no ostensible signs of mercurial affection.

12. She was ordered to take the following pill every eight hours: Submuriatis Hydrarg. gr. ij; Pulv. Opii, gr. $\frac{1}{3}$ m.

13. Inflammation of the right eye much the same, the left seems to partake of the affection.

14. The left eye inflamed and swollen; although she has taken but six of the pills, her mouth has become very sore, and salivation has taken place. Omit medicine.

15. The ptyalism is profuse; the tongue very much swollen, and projected from the mouth; the face tumid; the left eye very red and suffused with tears; she complains of constant pain in the head, with an aching, dragging sensation of the throat, consequent on the protruded tongue producing a tension of that part. In this condition the poor girl remained until the 2d of January, 1833. During this time it was found necessary to apply leeches twice to the eyelids, the left eye being then in a state of chemosis and very much swollen; and once to the tongue. She was also blistered, and had collyria of opium and argentum nitratum.

January 2. Mr. Lawrence, thinking to reduce the size of the tongue, which now presented a large round hard swelling, projecting from between the teeth, made an incision on either side. This occasioned great pain and aching of the part, which bled profusely, without affording any relief, or diminishing the size of the tongue. She passed a most distressing night, although she took five grains of the Pil. saponis c. opio. On the following morning Mr. Quin, house-surgeon, on examining the displaced organ, discovered that the tumefaction of the part, and the consequent pressure of the teeth above and below, had occasioned some ulceration, and also a strangulation, which, from the dry, black, and hard appearance it presented, led him to conclude would inevitably lead to sphacelation of the tongue, unless speedily relieved from its present constricted state. This he very judiciously endeavoured to effect by grasping the part with his hand, and gradually insinuating the tongue between the teeth, which were separated as much as possible; after some little time he succeeded in returning the whole of this immensely swollen organ to its natural situation in the mouth; having done this, he closed the jaws, and secured them in this position with a pasteboard splint and bandage.

4. Says she is "quite easy;" at first she experienced a great deal of pain after the tongue had been rescued from its perilous condition, but that as soon as it became moistened and warm, the swelling rapidly subsided. She can now articulate with facility, and take in nourishment, which beforehand she had found almost impracticable. Her eyes, especially the right, are very much improved; the left still is inflamed and sore, but looks a great deal better than it did some days since, when it presented almost a jelly-like appearance. The chemosis has disappeared.—Hyd. Nitrico Oxyd. gr. iss; Cerat. Cetacei, ʒi. M. to be smeared on the eyelids to prevent them sticking together, from the quantity of adhesive matter secreted by them.

8. Is better in all respects. The right eye seems quite restored to its natural state, and the left is only slightly inflamed; the tongue, although still sore from the incisions, has almost regained its normal condition. She expresses herself as very grateful to Mr. Quin for his exertions, to which alone she ascribes her present improved state.

This was one of those cases in which but a small quantity of mercury produced the most alarming effects. It clearly indicates the necessity that practitioners should ascertain the peculiar idiosyncrasies of their patients, previous to the administration of a drug, which sometimes, in its smallest doses, produces such deleterious consequences.—*Lancet.*

---

*Case of Retention of Urine and great Tumefaction of the external Parts, produced by a Calculus, impacted in the Urethra of a Child.*

John Jacques, a fine boy, aged four years, was brought to the hospital about nine o'clock on Friday morning, the 26th of October, and placed under the care of Mr. Earle: on his arrival, the whole scrotum and skin of the penis were found to be very œdematous, and much increased in size; the woman who accompanied him knew nothing further of the case than that the swelling had suddenly made its appearance early the same morning. Numerous scarifications were made with the point of a lancet, to relieve the excessive tension, after which, warm fomentations were continually applied. At two o'clock P.M., the tension had rather increased than diminished, notwithstanding that a considerable discharge of blood and serum had been kept up by the warm applications. On examination, no stricture of any kind, or other exciting cause, could be discovered; the affection was therefore attributed to irritation, produced by some acrid secretion under the prepuce, which was exceedingly œdematous, and in a state of phymosis. A weak solution of sulphate of zinc was ordered to be thrown up under the prepuce by means of a small India-rubber bottle.

At seven P.M., the boy having made no water since the morning, and the œdema having increased to a frightful extent, an attempt was made to pass into the bladder a very small elastic gum catheter, but without success: a slit about three fourths of an inch in length was then made in the upper part of the prepuce, but as the glans penis did not then make its appearance, some slight doubt arose as to the nature of a tumor, which resembled somewhat in shape and even in colour the glans penis in a state of disease: a slight puncture was made into it, but as nothing but pure blood oozed from the wound, no light was by this means thrown on the nature of the enlargement. The penis was again carefully examined, the œdema having somewhat diminished by the bleeding, &c.: a slight irregularity was discovered by pressing firmly the penis between the finger and thumb, at about three quarters of an inch from the pubis; and as this was the only irregularity felt, it was presumed to be the glans. The director was again inserted under the prepuce, and the incision continued to this point, when the tip of a small calculus was discovered at the orifice of the urethra, which was removed without much difficulty. In shape, the stone is a long square, about the third of an inch in length, a quarter in width, and too lines in thickness; one extremity forms a kind of wedge-shaped head, the very tip of which was the only part discoverable at the orifice of the urethra. A small elastic gum catheter was immediately introduced into the bladder, when about sixteen ounces of urine were drawn off: some further scarifications were then made in the prepuce and scrotum; a hip bath was ordered to be used immediately; and the parts to be enveloped in a bread and water poultice during the night.

27th. A considerable quantity of water is stated by the sister to have dribbled away during the night; and the boy appears to be doing well.

29th. Yesterday a small dose of castor oil was given. The water is made freely, but still in the bed. The belly this morning appears distended, but no external examination can give an exact idea of the state of the bladder, as the boy cries immediately on turning down the bed-clothes. The catheter has been introduced into the bladder, which was found to be empty. The scrotum is still swollen and much inflamed.—Ordered a hip-bath, and to continue the poultices.

30th. The scrotum still inflamed; has not passed much water during the night; the catheter was again introduced, and a large quantity of highly-coloured urine drawn off.—Repeat the bath and poultices.

31st. A considerable quantity of matter had formed around the root of the penis, which has been let out by a free incision: a large arterial branch was wounded, which required to be tied.

Nov. 2d. Another incision was made to-day on the opposite side of the root of the penis, as there was still a small collection of matter. The boy is rather blanched and weak.—Ordered to have meat diet, and a little beer.

6th. Since the 2d, no unfavorable symptoms have appeared; the boy now makes his water freely into a vessel, and none escapes through either of the wounds; in the urine there is a considerable deposit.

Fear was for some time entertained, from the suppuration in the cellular tissue of the scrotum, that ulceration of the urethra, and consequent effusion of urine, had taken place, from the irritation caused by the passage of the stone, and the introduction of the instruments; but the mischief has been so limited, that all fear on this head is dissipated, and especially as no water has passed through either of the wounds.

This case shows of how much importance it is to investigate minutely every case as soon as it presents itself to our notice, and of what value may be the statement of the friends, should the patient from age, the disease itself, or any other cause, be incapable of answering rationally those questions which it is necessary to put to him. Had the mother fortunately accompanied this little patient to the hospital, and given the subjoined statement, which she made the following Sunday morning, no doubt could have been entertained of the propriety of at once having recourse to those means which were resorted to ten hours afterwards, and thus the patient would have been saved much unnecessary pain and suffering, and the risk of his life.

The mother stated that on Thursday morning, (the morning previous to the boy's being brought to the hospital,) immediately after going to stool, he ran in, crying, and saying that "he had run a nail in.' As no wound was discovered, she thought nothing more of it, although he complained more or less of a pricking sensation during the rest of the day. The following morning, about six o'clock, on again going to stool, he again returned with the same exclamation: on examination the parts were discovered to be slightly swollen. The swelling increased very rapidly, as at nine o'clock he was brought to the hospital in the state above described. —*Med. Gazette.*

